# Self-Monitoring Home Blood Pressure in Community-Dwelling Older People: Age Differences in White-Coat and Masked Phenomena and Related Factors—The SONIC Study

**DOI:** 10.1155/2022/5359428

**Published:** 2022-04-30

**Authors:** Jinmei Tuo, Kayo Godai, Mai Kabayama, Yuya Akagi, Hiroshi Akasaka, Yoichi Takami, Yasushi Takeya, Koichi Yamamoto, Ken Sugimoto, Saori Yasumoto, Yukie Masui, Yasumichi Arai, Kazunori Ikebe, Yasuyuki Gondo, Tatsuro Ishizaki, Hiromi Rakugi, Kei Kamide

**Affiliations:** ^1^Department of Health Promotion System Sciences, Division of Health Sciences, Graduate School of Medicine, Osaka University, Suita, Japan; ^2^Department of Geriatric and General Medicine, Graduate School of Medicine, Osaka University, Suita, Japan; ^3^Department of General and Geriatric Medicine, Graduate School of Medicine, Kawasaki Medical School, Kurashiki, Japan; ^4^Department of Clinical Thanatology and Geriatric Behavioral Science, Graduate School of Human Sciences, Osaka University, Suita, Japan; ^5^Tokyo Metropolitan Geriatric Hospital and Institute of Gerontology, Tokyo, Japan; ^6^Center for Supercentenarian Medical Research, Keio University School of Medicine, Tokyo, Japan; ^7^Department of Prosthodontics, Gerodontology and Oral Rehabilitation, Graduate School of Dentistry, Osaka University, Suita, Japan

## Abstract

Some studies reported that home blood pressure (HBP) monitoring was conducted by community-dwelling older people themselves, but there have been few studies on HBP including very old populations aged over 90 years old. Thus, the aim of the present study was to clarify the current situation of white-coat and masked phenomena defined by on-site and home BP measurements in community-dwelling old and oldest-old populations. The study subjects were 380 participants from the SONIC study, a cohort study of a community-dwelling old population, who measured their HBP in a series of 3–5 days by themselves and brought their HBP records to the venue on the survey day. Study participants' characteristics were as follows: female, 185 (48.7%); male, 195 (51.3%); 70s, 95 (25.0%); 80s, 245 (64.5%); and 90s, 40 (10.5%). A total of 344 (90.5%) participants had hypertension. A total of 291 (76.6%) hypertensive participants taking antihypertensive medication were analyzed in the present study. Regarding the types of hypertension defined by home and on-site BP, they showed white-coat phenomenon, 183 (48.2%); masked phenomenon, 115 (30.3%); sustained hypertension, 130 (34.2%); and normotension, 82 (21.6%). On comparison of age groups, there was a tendency for the white-coat phenomenon to be common in young-old people in their 70s and the masked phenomenon to be common in very old people in their 90s. Therefore, since the detection of white-coat and masked phenomena is closely associated with appropriate BP management, it is very important for community-dwelling older populations to self-monitor HBP.

## 1. Introduction

According to the statistics Bureau of Japan, the proportion of the population aged 65 years old or older increased from 17.3% in 2000 to 28.7% in 2020, and it is expected to rise to 35.3% in 2040 in Japan [1]. Japan has become the most advanced super-aged society in the world.

Hypertension is one of the important factors of cardiovascular morbidity and all-cause mortality in individuals of all ages [2, 3], and there are often no subjective symptoms or signs, so it is called a “silent killer” [4]. A review from Kamide et al. reported that there was a higher CVD risk of elevated morning blood pressure (BP) in elderly patients with essential hypertension [5]. A study focusing on elevated SBP for 195 countries and regions from 1990 to 2015 reported that elevated systolic blood pressure (SBP) markedly increased disability-adjusted life years and deaths [6]. On the other hand, an inverted association was noted between SBP and geriatric syndrome, such as cognitive decline and frailty [7, 8]. Thus, the management of hypertension should be individualized in old people [7].

In the management of hypertension, out-of-office BP monitoring is used as an important monitoring tool, especially in the treatment of elderly hypertension [2]. Altered BP is common in old people [5], and their rates of white-coat hypertension and BP variability are higher than in younger adults. Individuals with masked hypertension have a higher prevalence of cardiovascular disease than those with controlled hypertension and white-coat hypertension among elderly people [9]. Even though the risk of white-coat hypertension is still controversial [10, 11], identification is important to avoid excessive hypotension and insufficient BP reduction caused by antihypertensive treatment [2, 3]. The measurement of home BP (HBP) is recommended because it is important for the continuation of hypertension treatment of the patient, and it is also helpful for physicians to be able to distinguish among normotension, white-coat phenomenon, masked uncontrolled hypertension, and sustained hypertension through HBP measurement [12].

However, studies on HBP monitoring focused on old people are limited. In previous studies on white-coat hypertension/phenomenon and masked hypertension/phenomenon, the average age of participants was around 50 years old. Particularly, there have been very few studies on super old people over 90 years. In addition, evaluation and treatment of hypertension should be individually considered because individual differences in the physiological function are marked in the elderly, even in those with the same age [2]. It is known that BP fluctuates widely in old populations. Therefore, in this study, we focused on what causes the difference in BP between on-site and home measurements.

The purpose of the present study was to clarify the current situation of white-coat and masked phenomena, defined by the difference in BP between on-site and home measurements, and associated factors in participants with hypertension taking antihypertensive medications in community-dwelling old and oldest-old populations.

## 2. Methods

### 2.1. Study Participants

This study was a part of a prospective observational population study, the Septuagenarians, Octogenarians, and Nonagenarians Investigation with Centenarians (SONIC) study, a study ongoing since 2010 [13]. The participants were all volunteers living independently, who were recruited from the residential registry and participate in the site survey near the residential area. The setting was four locations of urban and rural regions in Western and Eastern Japan. The SONIC study uses a narrow age range cohort design and has three age groups of people who are followed up: 69–71 years (70-year-old cohort), 79–81 years (80-year-old cohort), and 89–91 years (90-year-old cohort) every 3 years.

In the present study, the study subjects were 380 participants from the SONIC study who measure their HBP in a series of 3–5 days by themselves and brought their HBP records to the venue on the survey day. The 380 study subjects comprised 95 from the 70-year-old cohort (73 ± 1, 76 ± 1 year old), 245 from the 80-year-old cohort (80 ± 1, 83 ± 1, 86 ± 1 years old), and 40 from the 90-year-old cohort (90 ± 1, 93 ± 1 years old) participating in the SONIC study from 2012 to 2017.

The SONIC study was approved by the Institutional Review Board of Osaka University Graduate School of Medicine, Dentistry, and Human Sciences (Osaka, Japan) and the Tokyo Metropolitan Geriatric Hospital and Institute of Gerontology (Tokyo, Japan) (No. 14494 (306)-2). All participants provided written informed consent to participate.

### 2.2. Blood Pressure Measurements

On-site BP (OBP) was measured by nurses or doctors using a mercury sphygmomanometer or electronic monitor. OBP was measured twice using the left and right arms separately in a seated position with at least 1 minute of rest. The mean of the 2 measurements of both arms was used in the analysis.

Home BP (HBP) was measured by participants themselves for 3–5 days continuously in the morning at their home, and we copied the records that they brought. The participants measured and recorded BP by themselves using their own BP-measuring devices at home. In this study, we did not explain how to measure HBP, and we obtained BP data from daily BP recordings taken by participants as usual.

According to the criteria of the Japanese Society of Hypertension guidelines for the management of hypertension (JSH 2019) [2], hypertension was defined based on OBP values ≧ 140/90 mmHg or the use of antihypertensive medication. Information on medications was obtained from prescription records that the participants brought.

In the present study, we evaluated differences in SBP between on-site and the home (ΔBP). The white-coat phenomenon was defined as the on-site SBP minus home SBP >10 mmHg. The masked phenomenon was defined as the on-site SBP minus home SBP <0. Normotension was defined as the on-site SBP minus home SBP of 0 to 10 mmHg. Because of the 10 mmHg difference between on-site and home BP, there was no definitive evidence. Hence, we think a 10 mmHg difference may be appropriate since a 5 mmHg difference is usual, as described in the Japanese guideline (JSH2019) [2], and very few people with a 15 mmHg difference were seen in our study.

### 2.3. Definition of Other Factors

Medication information was obtained from the medication notebook. Even though we identified antihypertensive medications by type, since CCB is the most common antihypertensive medication and it suppresses blood pressure fluctuations, we discussed whether to take CCB.

Dyslipidemia was defined as when participants were taking hyperlipidemia medication, LDL ≥140 mg/dL and/or HDL ≤40 mg/dL. Diabetes mellitus was defined as when participants were taking diabetes mellitus medication, HbA1c ≥ 6.5 mg/dL and/or glucose ≥200 mg/dL. Body mass index (BMI) was calculated as the weight (in kilograms) divided by the square of the height (in meters).

We used the Japanese version of the Montreal Cognitive Assessment (MoCA-J) [14] to assess the participants' cognitive function by trained psychologists in the present study. The MoCA-J test is a brief cognitive screening tool that has greater sensitivity and specificity for detecting mild cognitive impairment (MCI) in community-dwelling elderly performing in the normal range than conventional cognitive tests. The MoCA-J total score is 30 points, with a higher cognitive function indicated by a higher score.

Carotid ultrasonography (GE LOGIQ Book X–P) was performed to measure intima-media thickening (IMT) of the common carotid artery (CCA) [15]. In this study, the mean value was defined as the mean carotid IMT (mean-IMT).

Information about smoking and current alcohol drinking was obtained based on a self-administered questionnaire.

### 2.4. Statistical Analysis

Descriptive data are summarized as *n* (%) or mean (SD). Differences in the clinical characteristics and BP profiles associated with BP categories were assessed using the analysis of variance and the chi-squared test in total participants and in those taking antihypertensive medications. The same analysis was performed for each age group. The proportions of each blood pressure category by age group were calculated and compared using the chi-squared test.

Logistic regression analyses were performed to investigate the factors associated with white-coat and masked phenomena. Model 1 was not adjusted for any variables, and model 2 was adjusted by sex, age group, BMI, MoCA-J, HDL-C, LDL-C, HbA1c, creatinine, mean IMT, diabetes mellitus, dyslipidemia, calcium channel blocker, on-site SBP, on-site DBP, smoking, and heavy alcohol drinking. The results are expressed as odds ratios (ORs) and 95% confidence intervals (CIs).

All statistical analyses were performed with SPSS Statistics 25.0 (IBM Japan Ltd., Tokyo, Japan). All reported *p* values are two-tailed, and *p* < 0.05 was considered significant.

## 3. Results

In the present study, total study subjects numbered 380, consisting of female, 185 (48.7%); male, 195 (51.3%); 70s, 95 (25.0%); 80s, 245 (64.5%); and 90s, 40 (10.5%). Regarding types of hypertension defined by home and on-site BP measurement, they showed: white-coat phenomenon, 183 (48.2%); masked phenomenon, 115 (30.3%); and normotension, 82 (21.6%). A total of 344 participants (90.5%) were determined to have hypertension. Of those, 291 participants (84.6%) were taking antihypertensive medication. Concerning the type of medication, the numbers of participants taking CCB were 222 (76.3%), ARB were 159 (54.6%), ACEI were 16 (5.5%), diuretics were 50 (17.2%), and *β* blocker were 44 (15.1%), and CCB takers made up the largest portion of those diagnosed with hypertension, shown in Supplementary Table 1.

The baseline characteristics for total and antihypertensive participants are presented in Table 1. We observed significantly different proportions of ΔΔSBP patterns (ΔSBP<0, 0≤ΔSBP≤10 mmHg, and ΔSBP>10 mmHg). The white-coat phenomenon pattern (ΔSBP>10 mmHg) was the most prevalent in the total subjects (48.2%) and antihypertensive subjects (45.5%). BMI and MoCA-J did not show significant differences in each group of ΔSBP among the total subjects. In addition, LDL-C, HT, and taking calcium channel blocker (CCB) showed significant differences among different groups of ΔSBP. Regarding the blood pressure, on-site SBP and on-site DBP were highest in the white-coat phenomenon group and home SBP and home DBP were highest in the masked phenomenon group.

Table 2 presents the current situation by age groups in the antihypertensive subjects. In those in their 70s, the 53.4% of subjects with white-coat phenomenon made up the largest group and 23.3% of subjects had masked phenomenon. In the group in their 80s, the white-coat phenomenon made up 43.7% and masked phenomenon made up 33.2%. Although we did not observe significance, we still found that subjects with the white-coat phenomenon were the most dominant (40.6%) in the group in their 90s. We observed the significance of both on-site SBP and home SBP among ΔSBP patterns in each age group.

Table 3 presents the number of participants by age groups with different ΔSBP patterns among antihypertensive participants. Although we did not observe a significant difference in ΔSBP patterns by age groups, the rate of the white-coat phenomenon was higher in those in their 70s while the rate of the masked phenomenon was higher in those in their 90s.

**Figure 1:**
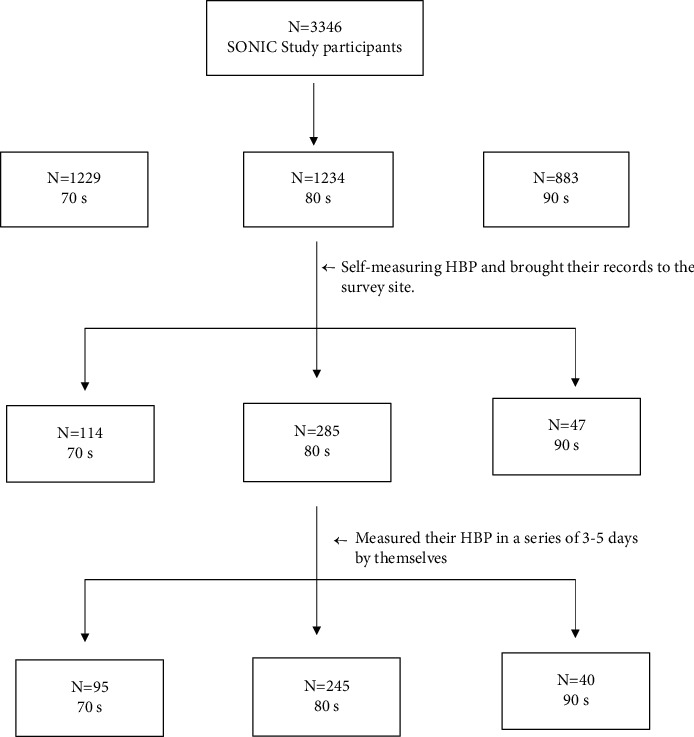
Participants' selection in the study.

Table 4 presents the odds ratios for both white-coat and masked phenomena in antihypertensive participants. In the unadjusted analysis (model 1), on-site SBP and on-site DBP were associated with white-coat and masked phenomena. After being adjusted by sex, age group, BMI, MoCA-J, HDL-C, LDL-C, HbA1c, creatinine, mean IMT, DM, dyslipidemia, CCB, on-site SBP, on-site DBP, smoking, and heavy alcohol drinking, the age group, CCB, on-site SBP, and on-site DBP were significantly correlated with the white-coat phenomenon and on-site SBP and on-site DBP were significantly correlated with the masked phenomenon (see Figure 1).

## 4. Discussion

In the present study, we analyzed 380 participants from community-dwelling older people who participated in the SONIC study. Among the total 291 subjects receiving hypertensive treatment, the rate of those with the white-coat phenomenon was 48.2% and that of those with the masked phenomenon was 30.3%. The present study showed that the white-coat phenomenon group was influenced by high on-site SBP and low home SBP in each age group. Multivariable results showed that those in their 70s tended to have the white-coat phenomenon and those in their 90s were more likely to have the masked phenomenon compared with those in their 70s.

Previous studies reported that the prevalence of white-coat hypertension was 13%, up to 35% [3, 16, 17], and also reported an increasing trend among the elderly [2]. There were differences from the present study. The reasons we considered were as follows. The definitions of BP were different. Furthermore, the age of study subjects in the previous study was different from that in the present study. The influence of age should be considered. Due to the dynamic characteristics of BP, white-coat hypertension should be different from the white-coat phenomenon. The white-coat phenomenon is defined as a high BP in the office or on-site but controlled BP at home in subjects with hypertension with or without taking antihypertensive medication [18, 19]. Therefore, the definition of white-coat hypertension according to ESC/ESH 2018 [20] is not fully applicable to the white-coat phenomenon. However, the results obtained in the present study are different from those of previous studies due to including older age subjects, so the rate of the white-coat phenomenon in our study may be relatively high. We think that the prevalence of the white-coat phenomenon in older people including very old subjects around 90 years is a novel finding.

On the other hand, Cacciolati's research on old populations over 65 years old living in three cities in France showed that 32% of those who did not receive treatment for hypertension and 49% of those who received treatment had masked hypertension [21]. Based on the JSH 2019 hypertension guideline, the prevalence of masked hypertension is 10–15% in normotensive residents and 9–23% of those with masked hypertension were reported to have a clinic BP controlled to less than 140/90 mmHg [2]. Furthermore, in the previous study of Spannella F et al., masked hypertension was noted in 10.8% and sustained hypertension was noted in 38.3% [22]. In that study [22], patients over 65 years old and with an average age of 71 years old were selected as the participants, and 24-hour ambulatory BP monitoring was used.

A total of 31.6% of those with masked phenomenon received hypertension treatment in our study. Moreover, based on the results of the present study, we found that the prevalence of the white-coat phenomenon is reducing and that of the masked phenomenon is increasing with aging in old populations. In the present study, community-dwelling residents over 70 years old were selected as the participants, and their HBP measured by themselves was used. The present study showed that the masked phenomenon has a higher prevalence in the group in their 90s.

Furthermore, previous studies by Cuspidi et al. showed that patients with masked hypertension show a stronger association with cardiovascular and cerebrovascular diseases than those with controlled hypertension [23]. Since HBP monitoring is common in Japan, self-home BP measurement is useful to clarify the white-coat or masked phenomenon, especially in old people. Self-monitoring of HBP can help physicians more accurately identify white-coat and masked phenomena. It is important to use HBP measurement as part of BP management for old and oldest-old populations.

The main cause of the white-coat phenomenon is considered to be activation of the autonomic nervous system [24]. Furthermore, structural alterations of the vascular system can lead to increased vascular resistance [25], and arterial tension may increase with age, causing arteriosclerosis. Thus, the white-coat phenomenon becomes more common in old people than in young and middle-aged people [5]. However, in general, autonomic nervous system activity is reduced in very old people, such as those aged 90 years. It may induce geriatric syndromes like syncope, orthostatic hypotension, and bradycardia in very old people [5]. In addition, night time high BP and a morning surge caused by a reduced glomerular filtration rate (GFR) [26] and congestive heart failure (CHF) [27] are more common in very old than young-old people. Therefore, we expected the white-coat phenomenon might be more common in those aged 70 years than in older people and the masked phenomenon to be more common in the very old, aged 90 years, than in young-old, aged 70 years.

Most previous studies showed that increases in various forms of hypertension-induced pathophysiological changes in old populations, leading to not only an increase of complications of cardiovascular and cerebrovascular diseases but also frailty, cognitive disorders, and other lifestyle-related diseases [28, 29]. Moreover, HBP monitoring may be useful to prevent these diseases. HBP measurement can be used to not only monitor the BP value but also effectively monitor the variation of BP [2, 3, 30].

From a review study, CCB was the most common antihypertensive medication [31]. CCB is an effective and safe antihypertensive medication for any age, especially old people [32, 33]. A previous study on the effect of antihypertensive medications on BP variability showed that CCB can reduce the daily average HBP significantly. Subjects who consented to CCB treatment showed a greater reduction in BP variability [34]. Moreover, a meta-analysis showed that CCB exhibits the strongest effect to reduce interindividual variation in SBP [35]. The results of the present study showed that the white-coat phenomenon readily develops without taking CCB. The effect of CCB on hypertension is relatively strong, resulting in higher HBP than in the venue. Therefore, it is particularly important to monitor daily BP with HBP.

To the best of our knowledge, the present study is one of the limited studies using self-measuring HBP to clarify the current situation of white-coat and masked phenomena in community-dwelling old people including those aged 90 years. We can discuss in-depth tracking and analysis of various chronic diseases and hypertension in old and very old people around 90 years. In addition, our study investigated factors related to white-coat and masked phenomena by adjusting for confounding factors associated with aging, including the cognitive function. Furthermore, it will be necessary to investigate the association between white-coat and masked phenomena and the onset of cardiovascular diseases or death in old and oldest-old populations by prospective observation in the future.

On the other hand, the present study also had some limitations. In the present study, due to the white-coat phenomenon defined by on-site SBP >10 mmHg, there may have been participants with hypertension in home blood pressure measurement. BP measurement in each subject was not uniform. There was a possibility of measuring in different seasons. Due to BP changes at any time, affected by position, temperature, and season, there may be some deviation in our results. The number of participants in each age group was not the same, so it may be difficult to generalize. The on-site BP and HBP may not have been determined in the same period, and hence, direct comparison may not be possible. Moreover, since the data of the present study used their self-measured BP, we could not perform validation of each subject's home BP device.

In conclusion, the white-coat phenomenon is common in community-dwelling old populations, especially the young-old around 70 years. The main cause of the white-coat phenomenon is considered to be activation of the autonomic nervous system [24]. Furthermore, progressed atherosclerosis in older people may lead to increased vascular resistance [25] and arterial tension may increase with age. Thus, the white-coat phenomenon is more common in old people than in young and middle-aged people [5]. However, autonomic nervous system activity reduces in very old people, such as those aged 90 years. In addition, night time high BP and the morning surge caused by reduced GFR [26] and combined CHF [27] are more common in very old people than young-old people. Therefore, the white-coat phenomenon might be more common in those aged 70 years than in older people, and the masked phenomenon might be more common in the very old, aged 90 years. In clinical practice, regardless of the presence of the masked or white-coat phenomenon, it is very important for community-dwelling older populations to self-monitor BP at home. Furthermore, self-monitoring BP at home may also be very important for maintaining fine cognitive function and ADL in oldest-old populations.

## Figures and Tables

**Table 1:** Results showing the current situation for total and antihypertensive participants.

	Total (*N* = 380)				Antihypertensive medicines (*N* = 291)			
	ΔSBP ＜ 0	0 ≦ ΔSBP ≦ 10	ΔSBP ＞ 10	*p*-value	ΔSBP ＜ 0	0 ≦ ΔSBP ≦ 10	ΔSBP ＞ 10	*p*-value
*N* (%)	115 (30.3)	82 (21.6)	183 (48.2)	^#^≤0.001	92 (31.6)	67 (23.0)	132 (45.5)	^#^≤0.001
BMI mean (SD)	22.57 (3.15)	23.26 (3.50)	22.88 (2.79)	0.309	22.73 (3.04)	23.65 (3.41)	22.76 (2.89)	0.109
MoCA-J mean (SD)	22.12 (4.48)	22.67 (4.19)	23.09 (4.19)	0.169	22.40 (4.51)	23.05 (3.74)	23.12 (4.22)	0.421
HDL-C mg/dL mean (SD)	60.78 (14.82)	59.90 (16.45)	60.68 (14.73)	0.911	60.51 (14.57)	59.22 (15.86)	60.98 (15.21)	0.744
LDLC mg/dL mean (SD)	110.96 (27.22)	108.23 (27.24)	118.26 (30.85)	0.017	110.34 (26.53)	107.99 (25.58)	115.62 (30.21)	0.154
HbA1c mean (SD)	5.89 (0.73)	5.89 (0.70)	5.81 (0.67)	0.494	5.92 (0.77)	5.85 (0.54)	5.79 (0.69)	0.393
Creatinine mg/dL mean (SD)	0.86 (0.27)	0.89 (0.48)	0.82 (0.26)	0.240	0.88 (0.26)	0.90 (0.52)	0.82 (0.26)	0.187
Mean IMT	0.85 (0.13)	0.85 (0.12)	0.84 (0.12)	0.882	0.85 (0.13)	0.85 (0.13)	0.85 (0.12)	0.940
HT (%)	98 (28.5)	71 (20.6)	175 (50.9)	^#^0.004	—	—	—	—
DM (%)	22 (19.8)	13 (16.5)	27 (15.1)	^#^0.575	18 (19.8)	9 (13.8)	18 (14.1)	^#^0.459
Dyslipidemia (%)	59 (52.2)	46 (57.5)	92 (50.5)	^#^0.582	50 (54.3)	40 (59.7)	66 (50.4)	^#^0.457
Antihypertensive medicines (%)	92 (82.1)	67 (82.7)	132 (72.1)	^#^0.059	—	—	—	—
DM medicines (%)	17 (15.1)	7 (8.6)	21 (11.5)	^#^0.674	15 (16.3)	5 (7.5)	14 (10.6)	^#^0.439
Dyslipidemia medicines (%)	37 (32.7)	33 (41.3)	60 (32.8)	^#^0.368	35 (38.0)	30 (44.8)	51 (38.6)	^#^0.643
Taking CCB (%)	72 (64.9)	55 (68.8)	95 (52.5)	^#^0.020	72 (78.3)	55 (82.1)	95 (72.5)	^#^0.289
Past smoking (%)	43 (39.4)	34 (43.0)	67 (38.1)	^#^0.754	36 (41.4)	29 (44.6)	46 (36.5)	^#^0.526
Current heavy drinking (%)	6 (5.4)	1 (1.3)	4 (2.2)	^#^0.182	6 (6.7)	1 (1.5)	3 (2.3)	^#^0.135
On-site_SBP mean (SD)	128.39 (12.73)	134.14 (10.82)	153.11 (15.42)	≤0.001	128.20 (12.78)	135.47 (10.30)	152.53 (13.90)	≤0.001
On-site_DBP mean (SD)	69.57 (10.00)	72.95 (9.59)	79.72 (11.00)	≤0.001	67.99 (8.79)	73.46 (9.15)	78.59 (10.26)	≤0.001
On-site_HR mean (SD)	71.25 (10.90)	70.80 (10.97)	70.79 (10.29)	0.930	70.58 (10.98)	70.31 (11.34)	71.59 (10.72)	0.677
Home_SBP mean (SD)	137.53 (11.82)	129.01 (10.28)	128.55 (12.31)	≤0.001	137.49 (11.61)	130.13 (9.90)	128.64 (10.94)	≤0.001
Home_DBP mean (SD)	86.31 (9.39)	83.60 (8.02)	83.58 (9.09)	0.026	85.90 (9.24)	83.68 (6.92)	83.26 (8.96)	0.069
Home_HR mean (SD)	68.97 (8.87)	68.55 (10.10)	67.13 (9.99)	0.287	68.70 (8.87)	67.65 (10.16)	66.93 (9.91)	0.445

BMI = body mass index; HDL-C = high-density lipoprotein cholesterol; LDL-C = low-density lipoprotein cholesterol; HbA1c = hemoglobin A1c; HT = hypertension; Mean IMT: mean intima-media thickness; DM = diabetes mellitus; CCB = calcium channel blockers; MoCA-J = Japanese version of Montreal Cognitive Assessment; SBP = systolic blood pressure; DBP = diastolic blood pressure; HR = heart rate; ΔSBP = SBP-Office-SBP-Home; SD = standard deviation. Statistical analysis: one-way analysis of variance, ^#^chi-square test.

**Table 2:** Current situation by age group in antihypertensive participants.

	70s (*N* = 60)				80s (*N* = 199)				90s (*N* = 32)			
	ΔSBP ＜0	0 ≦ ΔSBP ≦ 10	ΔSBP＞10	*p* value	ΔSBP ＜ 0	0 ≦ ΔSB*P* ≦ 10	ΔSBP＞10	*p*value	ΔSBP ＜ 0	0 ≦ ΔSB*P* ≦ 10	ΔSBP ＞ 10	*p*value
*N* (%)	14 (23.3)	14 (23.3)	32 (53.4)	^#^0.005	66 (33.2)	46 (23.1)	87 (43.7)	^#^0.002	12 (37.5)	7 (21.9)	13 (40.6)	^#^0.380
BMI Mean (SD) MoCA-J	23.29 (2.30) 23.93	24.74 (2.55) 24.79	23.32 (2.64) 24.34	0.193 0.836	22.40 (3.06) 22.50	23.76 (3.42) 22.50	22.75 (2.89) 23.33	0.067 0.353	23.89 (3.48) 19.82	20.78 (3.65) 23.20	21.53 (3.27) 18.33	0.119 0.170
Mean (SD) HDL-C mg/dL	(4.60) 60.79	(2.49) 62.58	(3.86) 52.83	0.019	(4.36) 61.33	(3.71) 58.46	(3.93) 63.76	0.177	(4.60) 55.67	(5.89) 58.43	(4.25) 60.85	0.721
Mean (SD) LDL-C mg/dL	(10.39) 114.33	(13.89) 112.32	(10.41) 114.46	0.977	(14.83) 108.23	(16.14) 106.58	(16.01) 114.48	0.188	(17.26) 117.32	(18.71) 109.86	(12.77) 125.71	0.605
Mean (SD) HbA1c	(34.88) 5.81	(25.16) 5.77	(28.48) 5.91	0.781	(25.52) 5.99	(27.43) 5.91	(27.24) 5.74	0.083	(20.90) 5.68	(11.07) 5.56	(48.82) 5.84	0.745
Mean (SD) Creatinine mg/dL	(0.65) 0.84	(0.51) 0.78	(0.69) 0.78	0.523	(0.81) 0.88	(0.56) 0.91	(0.64) 0.83	0.487	(0.67) 0.95	(0.42) 1.06	(1.04) 0.83	0.116
Mean (SD) Mean IMT	(0.17) 0.85	(0.16) 0.81	(0.19) 0.86	0.507	(0.28) 0.84	(0.61) 0.85	(0.29) 0.84	0.882	(0.26) 0.88	(0.23) 0.97	(0.22) 0.86	0.179
Mean (SD) DM (%)	(0.13) 3 (30.0)	(0.07) 1 (10.0)	(0.17) 6 (60.0)	^#^0.605	(0.13) 14 (43.8)	(0.13) 8 (25.0)	(0.10) 10 (31.3)	^#^0.273	(0.16) 1 (33.3)	(0.10) 0	(0.07) 2 (66.7)	^#^0.538
Dyslipidemia (%)	8 (21.6)	9 (24.3)	20 (54.1)	^#^0.885	36 (34.3)	28 (26.7)	41 (39.0)	^#^0.300	6 (42.9)	3 (21.4)	5 (35.7)	^#^0.843
DM medicines (%)	2 (28.6)	1 (14.3)	4 (57.1)	^#^0.822	12 (50.0)	4 (16.7)	8 (33.3)	^#^0.174	1 (33.3)	0	2 (66.7)	^#^0.524
Dyslipidemia medicines (%)	6 (22.2)	6 (22.2)	15 (55.6)	^#^0.952	26 (31.7)	22 (26.8)	34 (41.5)	^#^0.582	3 (42.8)	2 (28.6)	2 (28.6)	^#^0.751
Taking CCB (%)	9 (21.4)	11 (26.2)	22 (52.4)	^#^0.705	55 (34.6)	41 (25.8)	63 (39.6)	^#^0.051	8 (38.1)	3 (14.3)	10 (47.3)	^#^0.309
Past smoking (%)	5 (22.7)	6 (27.3)	11 (50.0)	^#^0.900	27 (34.2)	20 (25.3)	32 (40.5)	^#^0.665	4 (40.0)	3 (30.0)	3 (30.0)	^#^0.703
Current heavy drinking (%)	1 (50.0)	1 (50.0)	0	^#^0.264	4 (57.1)	0	3 (42.9)	^#^0.227	1 (100.0)	0	0	^#^0.409
On-site_SBP Mean (SD)	124.11 (8.47)	130.00 (8.88)	144.55 (11.56)	≤0.001	128.97 (12.76)	137.16 (9.90)	155.63 (13.96)	≤0.001	128.75 (16.71)	135.29 (13.04)	151.38 (11.17)	0.001
On-site_DBP Mean (SD)	70.00 (8.29)	75.30 (8.79)	77.56 (10.07)	0.050	67.86 (8.95)	73.80 (7.77)	80.23 (10.14)	≤0.001	66.42 (8.69)	67.50 (15.82)	70.13 (7.11)	0.645
On-site_HR Mean (SD)	68.29 (9.01)	69.11 (9.68)	66.47 (8.10)	0.596	70.77 (11.71)	70.85 (11.83)	73.07 (10.94)	0.384	72.25 (9.13)	69.21 (12.47)	74.35 (11.52)	0.607
Home_SBP Mean (SD)	134.68 (11.82)	124.48 (9.11)	125.92 (7.54)	0.006	136.78 (11.13)	132.12 (9.27)	129.92 (11.78)	0.001	144.70 (12.16)	128.34 (12.02)	126.71 (11.41)	0.002
Home_DBP Mean (SD)	87.85 (10.32)	82.62 (7.26)	83.34 (7.43)	0.167	84.92 (8.74)	84.60 (6.67)	84.04 (9.27)	0.815	89.04 (10.35)	79.73 (7.17)	77.83 (8.97)	0.013
Home_HR Mean (SD)	67.74 (6.22)	72.58 (11.96)	65.37 (9.82)	0.131	67.84 (8.97)	66.48 (9.21)	67.38 (10.37)	0.792	74.47 (9.66)	67.01 (12.39)	67.68 (6.26)	0.171

BMI = body mass index; MoCA-J = Japanese version of Montreal Cognitive Assessment; HDL-C = high-density lipoprotein cholesterol; LDL-C = low-density lipoprotein cholesterol; HbA1c = hemoglobin A1c; Mean IMT: mean intima-media thickness; DM = diabetes mellitus; CCB = calcium channel blockers; SBP = systolic blood pressure; DBP = diastolic blood pressure; HR = heart rate; ΔSBP = On-site_SBP-Home_SBP; SD = standard deviation. Statistical analysis: one-way analysis of variance, ^#^chi-square test.

**Table 3:** Number of participants by age group with different ΔSBP in antihypertensive participants.

	ΔSBP < 0 (*N* = 92)	0 ≦ ΔSBP ≦ 10 (*N* = 67)	ΔSBP>10 (*N* = 132)	*p*-value
70s *N* (%)	14 (23.3)	14 (23.3)	32 (53.4)	0.223
80s *N* (%)	66 (33.2)	46 (23.1)	87 (43.7)	0.568
90s *N* (%)	12 (37.5)	7 (21.9)	13 (40.6)	※0.223

ΔSBP = On-site_SBP-Home_SBP; statistical analysis: chi-square test. ※: ΔSBP < 0 vs. ΔSBP > 10.

**Table 4:** Standardized odds ratios for both white-coat phenomenon and masked phenomenon in antihypertensive participants.

	White-coat phenomenon		Masked phenomenon	
	Model 1 OR (95% CI)	Model 2 OR (95% CI)	Model 1 OR (95% CI)	Model 2 OR (95% CI)
Sex	1.518 (0.654–2.415)	1.787 (0.637–5.015)	0.669 (0.406–1.103)	0.624 (0.225–1.727)
Age group	0.751 (0.493–1.144)	0.382 (0.178–0.817)	1.431 (0.910–2.251)	1.470 (0.746–2.898)
BMI	0.962 (0.892–1.038)	0.962 (0.846–1.094)	0.965 (0.889–1.048)	0.956 (0.852–1.072)
MoCA-J	1.026 (0.970–1.086)	1.032 (0.942–1.130)	0.962 (0.908–1.019)	0.953 (0.872–1.041)
HDL-C mg/dL	1.004 (0.989–1.020)	0.991 (0.965–1.018)	1.001 (0.984–1.017)	1.005 (0.979–1.031)
LDL-C mg/dL	1.008 (1.000–1.017)	1.007 (0.994–1.020)	0.997 (0.988–1.006)	1.003 (0.990–1.015)
HbA1c	0.807 (0.568–1.147)	0.770 (0.348–1.706)	1.252 (0.881–1.780)	0.838 (0.405–1.734)
Creatinine mg/dL	0.441 (0.178–1.093)	0.214 (0.043–1.062)	1.387 (0.680–2.831)	1.387 (0.463–4.156)
Mean IMT	0.848 (0.133–5.426)	3.028 (0.142–64.610)	0.872 (0.119–6.376)	0.329 (0.017–6.201)
DM	0.782 (0.409–1.495)	1.679 (0.374–7.535)	1.516 (0.786–2.924)	2.366 (0.591–9.477)
Dyslipidemia	0.778 (0.489–1.2399	0.606 (0.277–1.326)	1.033 (0.629–1.697)	1.214 (0.581–2.537)
CCB	0.665 (0.385–1.147)	0.297 (0.116–0.762)	1.152 (0.637–2.083)	1.031 (0.441–2.411)
On-site_SBP	1.138 (1.105–1.172)	1.181 (1.128–1.236)	0.904 (0.880–0.928)	0.905 (0.875–0.936)
On-site_DBP	1.091 (1.061–1.121)	1.033 (0.987–1.081)	0.906 (0.878–0.935)	0.929 (0.886–0.973)
Past smoking	1.29980.800–2.109)	1.167 (0.453–3.007)	0.916 (0.547–1.535)	1.297 (0.511–3.294)
Current heavy drinking	0.500 (0.127–1.974)	1.155 (0.191–6.970)	3.434 (0.944–12.489)	4.832 (0.900–25.956)

BMI = body mass index; MoCA-J = Japanese version of Montreal Cognitive Assessment; HDL-C = high-density lipoprotein cholesterol; LDL-C = low-density lipoprotein cholesterol; HbA1c = hemoglobin A1c; Mean IMT = mean intima-media thickness; DM = diabetes mellitus; CCB = calcium channel blockers; SBP = systolic blood pressure; DBP = diastolic blood pressure; HR = heart rate; OR = odds ratio; CI = confidence interval. Statistical analysis: logistic regression. Model 1: no adjustment. Model 2: adjusted for sex, age group, BMI, MoCA-J, HDL-C, LDL-C, HbA1c, creatinine, mean IMT, HT, DM, dyslipidemia, CCB, On-site_SBP, On-site_DBP, past smoking, and current heavy drinking.

## Data Availability

The data based on the present study are only available for the member of researchers in the SONIC study because of the ethical issue.

## Supplementary Materials

Supplementary Materials Supplementary Table 1: the detail with antihypertensive medication
