# Supplementary material for: Self-Monitoring Home Blood Pressure in Community-Dwelling Older People: Age Differences in White-Coat and Masked Phenomena and Related Factors—The SONIC Study
Source: Int J Hypertens. 2022 Apr 30;2022:5359428. doi: 10.1155/2022/5359428 (PMC9078792; doi:10.1155/2022/5359428)
Supplement: Supplementary Materials — Supplementary Table 1: the detail with antihypertensive medication [file 5359428.f1.docx]

|  | Total  N=291 | 70S  N=95 | 80S  N=245 | 90S  N=40 | P-value |
| --- | --- | --- | --- | --- | --- |
| CCB  N(%) | 222(76.3) | 42(44.2) | 159(64.9) | 21(52.5) | 0.002 |
| ARB  N(%) | 159(54.6) | 31(32.6) | 109(44.5) | 19(47.5) | 0.119 |
| ACEI  N(%) | 16(5.5) | 3(3.2) | 9(3.7) | 4(10.0) | 0.148 |
| Diuretic  N(%) | 50(17.2) | 9(9.5) | 31(12.7) | 10(25.0) | 0.045 |
| Βblocker  N(%) | 44(15.1) | 11(11.6) | 27(11.0) | 6(15.0) | 0.754 |

Supplemental Table1 The detail with antihypertensive medication

CCB = calcium channel blockers; ARB: angiotensin receptor blocker; ACEI: angiotensin converting enzyme inhibitor.

Statistical analysis: chi square test.
